# Genome Wide Identification of Recessive Cancer Genes by Combinatorial Mutation Analysis

**DOI:** 10.1371/journal.pone.0003380

**Published:** 2008-10-10

**Authors:** Stefano Volinia, Nicoletta Mascellani, Jlenia Marchesini, Angelo Veronese, Elizabeth Ormondroyd, Hansjuerg Alder, Jeff Palatini, Massimo Negrini, Carlo M. Croce

**Affiliations:** 1 Data Mining for Analysis of Microarrays, Università degli Studi, Ferrara, Italy; 2 Department of Molecular Virology, Immunology and Molecular Genetics, Comprehensive Cancer Center, Ohio State University, Columbus, Ohio, United States of America; 3 Dipartimento di Medicina Sperimentale e Diagnostica, Centro Interdipartimentale di Ricerca sul Cancro, Università, Ferrara, Italy; 4 Institute of Cancer Research, Surrey, United Kingdom; Ordway Research Institute, United States of America

## Abstract

We devised a novel procedure to identify human cancer genes acting in a recessive manner. Our strategy was to combine the contributions of the different types of genetic alterations to loss of function: amino-acid substitutions, frame-shifts, gene deletions. We studied over 20,000 genes in 3 Gigabases of coding sequences and 700 array comparative genomic hybridizations. Recessive genes were scored according to nucleotide mismatches under positive selective pressure, frame-shifts and genomic deletions in cancer. Four different tests were combined together yielding a cancer recessive p-value for each studied gene. One hundred and fifty four candidate recessive cancer genes (p-value<1.5×10^−7^, FDR = 0.39) were identified. Strikingly, the prototypical cancer recessive genes *TP53*, *PTEN* and *CDKN2A* all ranked in the top 0.5% genes. The functions significantly affected by cancer mutations are exactly overlapping those of known cancer genes, with the critical exception for the absence of tyrosine kinases, as expected for a recessive gene-set.

## Introduction

A variety of approaches have been applied to the identification of cancer genes [1]. Procedures have been developed that allowed identification of genes causative of cellular transformation [2], [3], and of complex processes such as invasiveness and metastasis [4]. In vitro and in vivo methods, using cellular or animal models, led generally to the discovery of dominant cancer genes, or oncogenes. On the other hand, tumor suppressors have been discovered mainly by molecular genetics approaches. Such is the need of identifying additional tumor suppressors, or recessive cancer genes, that new tests for loss-of-function continue to be developed [5].

Many well-characterized cancer genes harbor somatic base substitutions or small insertion/deletions. For example, coding region frame-shifts and point mutations account for 75% of the somatic mutations in *CDKN2A* and *TP53*, two major tumor suppressor genes [6], [7], [8]. The oncogene *B-raf*, first described over 20 years ago, was also shown to be mutated in some human cancers [9], alongside *PI3K* and some tyrosine phosphatases [10]. Meanwhile, other cancer genes have been discovered through the phenomenon of inherited predisposition. Familial cancer is rare in comparison to non-hereditary cancer, but a number of recessive genes have been identified using linkage analysis [11], [12]. Large scale super-family sequencing projects, i.e. the kinome and phosphatome projects, followed and showed that, although missense mutations are found in some members of these two superfamilies, they are not a common ground for somatic cancer mutations. Greenman and co-workers [13] undertook comprehensive sequencing of 518 protein-kinase-encoding genes in 210 cancers. Kinases have been implicated in many aspects of tumorigenesis and several have now been validated as targets for drug therapy [14]. In their analysis of the collection of cellular kinases, the kinome, Greenman et al. [13] identified 1,000 mutations. Mutations were relatively common in cancers of the lung, stomach, ovary, colon and kidney, and rare in cancers of the testis and breast, and in carcinoid tumors, which are usually found in the gastrointestinal tract. Tumors with defects in DNA-mismatch repair harbored large numbers of mutations, whereas other types of tumor revealed no detectable mutations. To distinguish driver from passenger mutations, Greenman et al. used a statistical model comparing the observed-to-expected ratio of synonymous (no amino-acid change) mutations with that of non-synonymous (altered amino acid) mutations. An increased proportion of non-synonymous mutations implies selection pressure during tumorigenesis. Overall, they identified 158 predicted driver mutations in 120 kinase genes. In contrast to the recurrent mutations in *BRAF* in malignant melanomas [15] most kinase mutations identified across different tumor types were therefore single hits. More recently, Wood and co-workers [16] used a different strategy, but reached similar conclusions, with the complete sequencing of 20,857 transcripts from 18,191 genes in a limited number of tumors (11 breast and 11 colon). The high number of automatically detected DNA mutations provided immediately the following question: how to identify from a potentially high number of sequence mismatches those that are causative of cancer pathogenesis. A series of subsequent filters revealed that most of them were silent (did not result in amino acid change) and a similar amount were single nucleotide polymorphisms (SNPs). The final number of mutations which were defined as truly somatic affected more than 1000 genes. Interestingly, few common driver mutations were identified among the kinase genes in these studies. This is consistent, for example, with the finding that only 1 out of 18 members of the *PI3K* family had somatic mutations in cancer [17].

Interesting observations can be made from an accurate global study of the mutations reported in cancer. Futreal et al. [18] conducted such an extended census from bibliography indicating that as many as 299 genes contribute to human cancer. However 70% of these genes are associated with leukemias, lymphomas and mesenchymal tumors, which account for only 10% of cancer incidence. Furthermore about 75% of those genes are associated with translocations, and at least 90% of listed cancer genes are dominant at the cellular level (i.e. activated oncogenes, fusion oncoproteins). Nevertheless, it is generally recognized that the vast majority of germline mutations resulting in cancer predisposition are recessive [18]. Thus it seems likely that most of the cancer genes are recessive and remain still undiscovered.

For these reasons we devised a novel method for the identification of candidate recessive cancer genes from genome-scale datasets. We applied our novel procedure to mine data from sequences and comparative genomic hybridizations. Our method takes account of the different gene inactivation modes, ranging from point mutations to whole gene deletions. The assumption underlying our investigation was that, by studying cancer genes from different mutational perspectives and combining the respective probabilities, sequencing noise and polymorphisms could be filtered out and bona fide recessive cancer genes would be identified.

## Results

### Harvesting candidate mutations from ESTs

**Figure 1:**
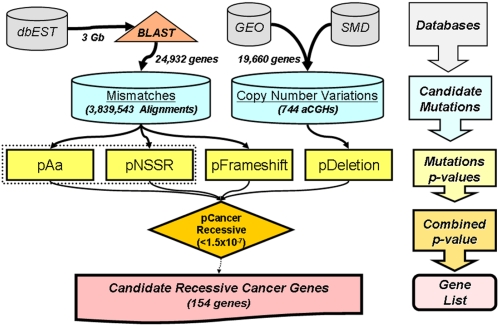
The rationale for selection of candidate recessive cancer genes. The diagram shows the steps in the procedure for the evaluation of mutation probabilities and the data flow towards the identification of candidate recessive cancer genes. Molecular data were extracted from public databases (dbEST and GEO at NCBI, and Stanford Microarray Database). A very large number of alignments (over 4.5 million) was obtained for over 24,000 human genes from BLAST analysis of 3 Gbases of EST sequences. The alignments were parsed to extract mismatches which were deposited in the Cancer Mutome local SQL database. The mismatches were then evaluated by specific procedures to associate mutational p-values to each human gene. In parallel, almost 20,000 human genes were assayed from 744 array CGH to define their propensity to deletion in cancer. The specific mutational p-values were combined to produce a recessive cancer p-value. A genome subset of 154 genes, among which *TP53*, *PTEN*, *CDKN2A* and *CDKN2B* were present, was selected (cancer p-value<1.5×10^−7^).

In this paper, a novel method was applied to the identification of genes mutated in non-hereditary human cancers (Figure 1). The procedure gathered sequence information from the expression sequence tag (EST) database and an appropriate algorithm was tailored to extract information from “low quality” sequence data. The procedure analyzed more than 3×10^9^ nucleotides of human coding sequence in over 5,600,000 ESTs derived from both healthy and cancerous tissues and cell lines. ESTs are potentially very valuable for mutation studies since they represent cloned single alleles, but are also unverified sequences, with a high rate of sequencing errors [19], [20]. Therefore, in order to exploit the full potential of ESTs we had to develop a method for the detection of bona fide “cancer” mutations in a context of frequent sequencing errors or, at best, polymorphisms. Although previous work [19] attempted to evaluate sequencing error rate in ESTs, we followed an alternate route. Our procedure was based on the assumption that the rate of sequencing errors was constant for each human gene, at each nucleotide position. As a corollary, we assumed that the “gene/position-specific sequencing error rate” was constant across normal and cancer EST libraries. Since base composition, context and sequence are by definition constant within each different human gene, we believed these assumptions were safe. Only exceptions would be due to the tumors harboring DNA repair defects.

High sequencing noise was expected to be present in the heterogeneous EST database and cancer is a complex multi-faceted genetic disease, therefore a single statistical test would not result in reliable selection of cancer genes. Furthermore, we wanted to focus on recessive genes, inactivated by the occurring events. Thus, to assay the different mutational modes of recessive cancer gene, we accordingly devised a number of mutational tests. The statistical tests were eventually combined to identify the genes that are often inactivated in cancer.

Starting from the RefSeq human mRNA repository, 27,184 sequences (defined Queries) were aligned to more than 5.6 million human EST sequences, from 7574 different EST libraries, for a total of almost 3.0 Gbases of coding sequence. BLASTs [21] were run for each query versus the ESTs and 3,839,543 successful alignments were produced (stored in the Alignments SQL table of the Cancer Mutome database) for 24,932 human queries (Stats database table). An average of 150 hits (high scoring pairs, HSP. or sequences) was produced for each query (human gene or splicing variant). The quality control of the BLAST alignments was of the foremost importance for our strategy. In order to minimize the mining of technical errors we defined a stringent threshold for alignment quality (expect≤1E-21) and the low quality ends of alignments were discarded. All (43,965,904) nucleotide mismatches, and gaps/insertions, were recorded in the database Mutations table. Amino acid (AA) substitutions and premature stops (33,614,754 mismatches) were then selected from the alignments (AA_Mutation table). To reduce the complexity, and the expected number of false positives, we decided to evaluate only those genes with a high number of mismatches (irrespective of the samples cancer status). A pre-processing based on inter-quartile range (IQR) was therefore applied and 8,972 genes (IQR higher than 0.5) were retained for further cancer mutation assays. These genes were sufficiently rich in putative mutations (mismatches) to fulfill the role of potential cancer gene candidates.

The first component of our strategy was the identification of genes harboring inactivating point mutations. We evaluated the point mutations according frequency, location, capacity to alter the amino acid sequence, and consequences on the reading frame. Our procedure was thus tailored to consider statistically all the above features of a point mutation.

### Data mining for amino-acid substitutions and premature terminations

We defined pAA as the probability that a gene displays an excess of amino acids substitutions in cancer when compared to non cancer samples. pNSSR, instead, indicates the probability that the significant amino acids substitutions in the cancer samples are under positive selection pressure. To detect short range clustering of cancer mutations, common in cancer recessive genes, and to balance out noise, i.e. sequencing errors, we chose a paired t test coupled to a sliding window. We normalized the counts of the mismatches in the two classes, cancer and control, by using a gene specific and position specific factor. Null mismatch counts were adjusted to unity, prior to normalization. The normalization values were obtained, for each gene and at each nucleotide position, as the local ratios of the sequenced nucleotides in the cancer and control samples. The paired t test (cancer vs. control, paired for codons) was applied to a sliding window with a length of 25 codons. To perform a robust assay a codon was evaluated only when aligned at least 10 times in each class (cancer and control). Gene specific confidence limits for T scores where generated by bootstrap analysis and a threshold p-value of 0.05 was used to select the significant amino acid positions. For each human gene, a p-value (pAA) was finally associated to the sum of the peaks corresponding to the significant T scores. A sequence mismatch was recorded only once for each EST library.

An over-estimation of pAA could be due to passenger mutations, such as those produced by altered DNA repair systems, prevalent in some cancer. Since passenger mutations should be randomly distributed over the genome, an additional test was therefore implemented to refine the pAA. The ratio of non-synonymous (NS) to synonymous (S) DNA mutations is a measure of the selective pressure during tumor progression, as synonymous alterations are unlikely to exert a growth advantage and will be selectively lost [17]. Furthermore, mismatches due to sequencing errors, as well as differential representation (cancer to normal differential expression), are all expected to be neutral with respect to the NS to S ratio. The codons significant for amino acid substitutions (p<0.05) were therefore assayed for positive pressure. As a proof-of-concept, the NS/S ratios in the *TP53* mutated region were analyzed by paired t test (p<0.033, FDR = 0.092) and revealed higher values in cancer than in control. Thus we applied the NS to S ratio test to each gene, in cascade after that for the local mutation frequency (pAA) described above. Bootstrap was again used to define the p-values. The probability of a cancer protein having frequent amino acid changes (pAA) coupled to selective positive pressure in cancer (pNSSR), two events which are not independent, was defined as the average of the two respective p-values (pAA-NSSR).

### Data mining for frame-shifts in cancer ESTs

Having defined for each human gene a p-value for causal amino acid substitutions in sporadic cancers, we needed a corresponding index for gene inactivation due to open reading frame shifts in exons. Cancer genes can be disrupted by micro-insertions or -deletions in their coding sequence, resulting in an altered primary structure. A genome wide survey of our mismatch database indicated that single nucleotide alterations were by far the most common insertions/deletions in ESTs. We indicated with pFrameshift the probability that a gene had an excess of frame-shifts, due to single nucleotide deletions/insertions in cancer, compared to control tissues. We tested the hypothesis that these mutations were frequent in cancer genes, by studying again *TP53*. Our assay showed that single nucleotide frame-shifts associated to cancer were non-randomly enriched in *TP53*. When looking for frame-shifts induced by 1 nucleotide insertions/deletions, an analogous test to that for pAA was designed, as detailed in Experimental Procedures, to generate pFrameshift.

### Identification of deleted genes in cancer by high resolution array comparative genomic hybridization

Cancer genes can be affected in their genomic structure by large amplifications and deletions. Recessive cancer genes are expected to be deleted or otherwise inactivated and this component must be included in our mutational model. We therefore assigned to each human gene p-values for deletion in cancer. To obtain such p-values, we compiled data from high resolution comparative genomic hybridizations of 744 tumors into the GeoSoft database. We used array CGH (aCGH), obtained from GEO (NCBI) and SMD (Stanford Microarray Database), with sufficiently high resolution to distinguish the human genes (information for samples and datasets in supplemental Table S1). Each tumor sample was compared to a healthy control sample on a two channel oligonucleotide-based platform. The human genes were evaluated in each sample by using the normalized log2 ratio (tumor over control). Different probes related to the same gene were averaged. Gene symbols were used as keys to unequivocally identify a gene within and across platforms. Data were normalized according to the providers. As a pre-processing step we reduced the assay complexity by retaining only those genes with high variability (standard deviation of log_2_ ratio>0.2). Then, for each gene we computed the percentiles of the log_2_ ratios (only for genes measured in at least 300 samples). A gene affected by deletions in tumors would possess a low (negative) log_2_ ratio 5^th^percentile, while one with amplifications would display a high (positive) 95^th^ percentile.

Bootstrap analysis (random swap between the tumor and control channels) was used to simulate gene specific 5^th^ and 95^th^ percentiles. Then, gene specific p-values for deletions (pDeletion) were finally calculated as the percentage of simulated 5^th^ percentiles exceeding the real 5^th^ percentiles. At this stage, we had to take in consideration two phenomena, associated to aCGH but not linked to cancer: sex chromosomes and polymorphic structural copy number variations (CNVs). The control sample in aCGHs was frequently from male (more than 50% of aCGHs), while roughly half of the tumors were of female origin and thus lacked the Y-chromosome. Therefore the Y-chromosome genes were expected to appear as deleted, or better “pseudo-deleted”. Conversely, we expected the X chromosome genes, except for those belonging to the pseudo-autosomal region, to appear as “pseudo-amplified”. Genes located in the sex chromosomes indeed behaved correctly, as shown in detail for the pseudo-autosomal region 1 (PAR1) in Xp22 (supplemental Figure S1). Polymorphic CNVs, from normal population variability and not linked to cancer, should also lead to large fold-changes, resulting in high 95^th^ or low 5^th^ percentiles. However, we expected that polymorphic CNVs, not associated to cancer, would not display significant pDeletion values. In fact their 5^th^ percentiles would not qualify as significant after the random swap simulation. *CDKN2A* and *CDKN2B* were identified as the most deleted genes in human cancers; *PTEN*, *ATM*, and *TP53* were also identified as deleted (p-values<0.001). Three thousand and three hundred seventy four genes were significantly deleted (p<0.001).

### Combination of mutation analyses: the candidate recessive cancer genes

Cancer genes are affected by different types of point mutations and of chromosomal alterations. We defined a candidate cancer gene as recessive when affected by mutations potentially leading to loss of function; i.e. when it was frequently mutated in its coding region and frequently altered in its genomic structure, in particular deleted. The combination of the different genome wide tests produced a p-value for recessive cancer genes. The recessive cancer gene (pRecessiveCancer) p-value was defined as the product of the three p-values (pAA-NSSR, pFrameshift, pDeletion). One hundred and fifty four human genes were included in the final candidate gene list after combinatorial mutation analysis was performed (pRecessiveCancer<1.5×10^−7^). The number of cancer recessive genes in a simulation by random association of the four mutation tests was of 60.5 (false detection rate of 0.39). The selection by the combinatorial approach appeared to be specific, since three classical recessive cancer genes, *TP53* (16^th^ position), *PTEN* (92^nd^) and *CDKN2A* (135^th^) were detected. When we compared the candidate gene-set to the whole genome, no major bias emerged towards gene size and structural polymorphisms, as expected from a well-behaved statistical procedure. The recessive cancer gene sizes did not differ significantly from that of the whole human genome (supplemental Figure S2). When we considered copy number variations, the cancer gene-set contained 15 polymorphic CNVs (15/154 or 10%) while 13.6% of all genes scored for pDeletion contained at least one CNV. This difference in proportion was not significant (p>>0.05), suggesting that there was no false enrichment for CNVs by our method, as expected by the design of the algorithm.

### Gene ontology and functional analysis

The mechanisms and functional pathways associated with the cancer recessive genes were statistically evaluated. The enrichment in Gene Ontology (GO) terms was assessed by using EASE, at http://david.abcc.ncifcrf.gov. The biological processes significantly affected in the cancer gene set are listed in supplemental Table S2. The significant GO terms grouped by EASE functional clustering were: ATP/nucleotide binding, cell death/apoptosis, cell cycle, mitochondrion, RNA binding, methylation, tumor suppressor, DNA metabolism and DNA repair (EASE enrichment score >2, EASE P-value<1×10^−4^, Benjamini p-value<0.01). A highly overlapping functional spectrum was obtained for the Cancer Census genes [18]. The most notable exceptions to the overlapping ontologies in the two cancer gene-sets were related to “protein tyrosine kinases”, absent from the candidate recessive list. These proteins are one of the most represented classes of oncogenes, or dominant cancer genes. A functional classification similar to that of EASE was obtained with BinGO and Cytoscape (data not shown), where some of the most significant cellular processes identified were involved in cancer pathogenesis, such as cell cycle, cell death/apoptosis (corrected p-value<1×10^−3^). Finally, we generated a control set of human genes by random associating the p-values from the four mutation tests. When EASE and BinGO were applied to this control set no significant GO terms were identified.

## Discussion

We devised and applied a multi-tier genome-wide data mining assay towards the identification of genes prone to “recessive-type” mutations in cancer. The p-values resulting from each tier were combined to produce a “recessive cancer gene” p-value (Table 1 and 2). Three of the most notable cancer recessive genes, i.e. *TP53*, *PTEN* and *CDKN2A*, ranked 16^th^, 92^nd^ and 135^th^, respectively, among all tested human genes. The block diagram of our rationale and the data flow are shown in Figure 1. The tests can be subdivided into two groups: one for detection of point mutations (amino acid substitutions and frame-shifts) and one for structural alterations (large deletions). In principle we could have also used a test for partial gene deletions, but in ESTs intra-gene rearrangements can be confounded with alternative exon splicing.

**Table 1:** Mutation p-values for the candidate recessive cancer genes.

GENE SYMBOL	pDeletion	pAA	pNSSR	pAA-NSSR	pFrameshift	pRecessive Cancer
*NASP*	5.00E-05	0.0005	0.0005	0.0005	0.0005	1.25E-11
*CCNB1*	0.0001	0.0005	0.0005	0.0005	0.0005	2.50E-11
*DDX21*	0.0002	0.0005	0.0005	0.0005	0.001	1.00E-10
*DHX9*	5.00E-05	0.0005	0.0005	0.0005	0.004	1.00E-10
*GANAB*	5.00E-05	0.0005	0.0005	0.0005	0.004	1.00E-10
*ILF3*	0.0005	0.0005	0.0005	0.0005	0.0005	1.25E-10
*AIPL1*	5.00E-05	0.002	0.011	0.0065	0.0005	1.63E-10
*NOLC1*	5.00E-05	0.004	0.003	0.0035	0.001	1.75E-10
*MYO1C*	5.00E-05	0.004	0.014	0.009	0.0005	2.25E-10
*NUDC*	0.0012	0.0005	0.0005	0.0005	0.0005	3.00E-10
*PGAM1*	0.002	0.0005	0.0005	0.0005	0.0005	5.00E-10
*IPO4*	0.0003	0.003	0.0005	0.0018	0.001	5.25E-10
*XRCC5*	5.00E-05	0.0005	0.0005	0.0005	0.021	5.25E-10
*MTO1*	5.00E-05	0.0005	0.0443	0.0224	0.0005	5.60E-10
*ANP32B*	5.00E-05	0.006	0.0421	0.0241	0.0005	6.02E-10
***TP53***	5.00E-05	0.022	0.031	0.0265	0.0005	6.63E-10
*AFG3L2*	5.00E-05	0.013	0.002	0.0075	0.002	7.50E-10
*FAF1*	5.00E-05	0.0737	0.006	0.0398	0.0005	9.96E-10
*CALR*	0.002	0.0005	0.0005	0.0005	0.001	1.00E-09
*SREBF2*	0.004	0.0005	0.0005	0.0005	0.0005	1.00E-09
*XRCC6*	5.00E-05	0.007	0.002	0.0045	0.005	1.12E-09
*ARMC8*	5.00E-05	0.002	0.0005	0.0013	0.02	1.25E-09
*GTPBP4*	5.00E-05	0.005	0.002	0.0035	0.008	1.40E-09
*HSPA4*	0.0004	0.016	0.001	0.0085	0.0005	1.70E-09
*HDAC1*	5.00E-05	0.001	0.0005	0.0008	0.0486	1.82E-09
*PGD*	5.00E-05	0.075	0.0005	0.0378	0.001	1.89E-09
*VCP*	0.002	0.0005	0.0005	0.0005	0.002	2.00E-09
*ATXN2L*	0.0025	0.0005	0.0005	0.0005	0.002	2.50E-09
*RPL6*	0.001	0.001	0.009	0.005	0.0005	2.50E-09
*SARS*	5.00E-05	0.0952	0.007	0.0511	0.001	2.56E-09
*NCL*	0.0001	0.005	0.001	0.003	0.01	3.00E-09
*PTPRC*	0.012	0.0005	0.0005	0.0005	0.0005	3.00E-09
*SMARCA4*	0.012	0.0005	0.0005	0.0005	0.0005	3.00E-09
*CCT3*	0.0004	0.012	0.004	0.008	0.001	3.20E-09
*NET1*	5.00E-05	0.01	0.001	0.0055	0.013	3.58E-09
*HNRPD*	5.00E-05	0.011	0.0005	0.0057	0.013	3.74E-09
*SQSTM1*	0.01	0.001	0.0005	0.0008	0.0005	3.75E-09
*TUBB2C*	0.002	0.0005	0.007	0.0037	0.0005	3.75E-09
*C1QBP*	0.002	0.001	0.007	0.004	0.0005	4.00E-09
*TRAP1*	0.002	0.0005	0.0005	0.0005	0.004	4.00E-09
*ALDOA*	0.018	0.0005	0.0005	0.0005	0.0005	4.50E-09
*RNASEH2A*	5.00E-05	0.1183	0.0651	0.0917	0.001	4.59E-09
*DDX24*	0.002	0.002	0.0005	0.0013	0.002	5.00E-09
*ILVBL*	5.00E-05	0.019	0.001	0.01	0.01	5.00E-09
*SERPINB3*	5.00E-05	0.1205	0.2837	0.2021	0.0005	5.05E-09
*UQCRC1*	5.00E-05	0.016	0.0005	0.0083	0.016	6.60E-09
*EEF2*	0.028	0.0005	0.0005	0.0005	0.0005	7.00E-09
*NUSAP1*	5.00E-05	0.001	0.008	0.0045	0.033	7.43E-09
*DNAJC11*	0.0002	0.1653	0.008	0.0866	0.0005	8.66E-09
*HSP90AA1*	0.036	0.0005	0.0005	0.0005	0.0005	9.00E-09
*MYH9*	5.00E-05	0.0709	0.002	0.0365	0.005	9.12E-09
*HK1*	5.00E-05	0.01	0.001	0.0055	0.034	9.35E-09
*IARS*	0.01	0.003	0.001	0.002	0.0005	1.00E-08
*YBX1*	0.004	0.0005	0.0005	0.0005	0.005	1.00E-08
*HDLBP*	0.03	0.0005	0.001	0.0008	0.0005	1.12E-08
*EWSR1*	0.02	0.0005	0.002	0.0013	0.0005	1.25E-08
*DHX15*	5.00E-05	0.0456	0.0005	0.023	0.011	1.27E-08
*SERPINB4*	5.00E-05	0.3571	0.6667	0.5119	0.0005	1.28E-08
*POLR2A*	5.00E-05	0.038	0.005	0.0215	0.012	1.29E-08
*ALG14*	5.00E-05	0.098	0.1023	0.1002	0.003	1.50E-08
*PRMT1*	0.002	0.003	0.012	0.0075	0.001	1.50E-08
*COX4NB*	5.00E-05	0.001	0.005	0.003	0.1047	1.57E-08
*SPTBN1*	5.00E-05	0.026	0.004	0.015	0.021	1.58E-08
*PTPRF*	5.00E-05	0.0455	0.0005	0.023	0.014	1.61E-08
*KHDRBS1*	5.00E-05	0.117	0.013	0.065	0.005	1.62E-08
*PABPC1*	0.002	0.0005	0.003	0.0018	0.005	1.75E-08
*CTNNA1*	0.018	0.0005	0.0005	0.0005	0.002	1.80E-08
*DDB1*	0.018	0.0005	0.0005	0.0005	0.002	1.80E-08
*GNB2L1*	0.074	0.0005	0.0005	0.0005	0.0005	1.85E-08
*WDR1*	0.002	0.003	0.001	0.002	0.005	2.00E-08
*AARS*	0.024	0.003	0.0005	0.0018	0.0005	2.10E-08
*NDE1*	0.0001	0.012	0.002	0.007	0.03	2.10E-08
*NQO1*	0.002	0.002	0.0005	0.0013	0.009	2.25E-08
*RUVBL2*	5.00E-05	0.006	0.1778	0.0919	0.005	2.30E-08
*ZWINT*	5.00E-05	0.0496	0.003	0.0263	0.018	2.37E-08
*HP1BP3*	0.0007	0.002	0.006	0.004	0.009	2.52E-08
*WDR79*	5.00E-05	0.0501	0.002	0.026	0.02	2.60E-08
*SLC25A6*	0.002	0.0005	0.005	0.0027	0.005	2.75E-08
*TYMS*	5.00E-05	0.037	0.009	0.023	0.024	2.76E-08
*SLC25A3*	0.06	0.0005	0.0005	0.0005	0.001	3.00E-08
*ACLY*	5.00E-05	0.0798	0.02	0.0499	0.014	3.49E-08
*ALDH3A1*	0.14	0.0005	0.0005	0.0005	0.0005	3.50E-08
*TTC8*	5.00E-05	0.015	0.1626	0.0888	0.008	3.55E-08
*YME1L1*	5.00E-05	0.0403	0.015	0.0276	0.026	3.59E-08
*ATP5A1*	5.00E-05	0.029	0.008	0.0185	0.039	3.61E-08
*MRPS2*	5.00E-05	0.007	0.0915	0.0493	0.015	3.69E-08
*HNRPH3*	5.00E-05	0.0816	0.0005	0.0411	0.018	3.70E-08
*IMMT*	0.004	0.038	0.004	0.021	0.0005	4.20E-08
*IMPDH2*	0.006	0.014	0.0005	0.0073	0.001	4.35E-08
*NCKAP1*	5.00E-05	0.0417	0.0745	0.0581	0.015	4.36E-08
*TTLL12*	5.00E-05	0.019	0.01	0.0145	0.0606	4.39E-08
***PTEN***	0.002	0.0721	0.017	0.0445	0.0005	4.45E-08
*WBSCR16*	0.182	0.0005	0.0005	0.0005	0.0005	4.55E-08
*XPNPEP1*	5.00E-05	0.0926	0.0005	0.0465	0.02	4.65E-08
*SREBF1*	5.00E-05	0.0651	0.3175	0.1913	0.005	4.78E-08
*CCDC5*	5.00E-05	0.0907	0.005	0.0479	0.021	5.02E-08
*DDX19B*	5.00E-05	0.007	0.0005	0.0037	0.2685	5.03E-08
*MAPK6*	5.00E-05	0.0692	0.2286	0.1489	0.007	5.21E-08
*MAP4*	5.00E-05	0.0442	0.0005	0.0223	0.0469	5.24E-08
*PHB2*	0.22	0.0005	0.0005	0.0005	0.0005	5.50E-08
*SAE1*	0.016	0.0005	0.0005	0.0005	0.007	5.60E-08
*TALDO1*	5.00E-05	0.1008	0.063	0.0819	0.014	5.73E-08
*AHCY*	0.23	0.0005	0.0005	0.0005	0.0005	5.75E-08
*GTF3C1*	0.0001	0.0496	0.001	0.0253	0.023	5.82E-08
*PRPF19*	0.002	0.0549	0.005	0.0299	0.001	5.99E-08
*LASP1*	5.00E-05	0.0522	0.007	0.0296	0.0409	6.06E-08
*TRIP10*	5.00E-05	0.1418	0.01	0.0759	0.016	6.07E-08
*HSPD1*	0.244	0.0005	0.0005	0.0005	0.0005	6.10E-08
*EIF4G2*	0.016	0.0005	0.015	0.0077	0.0005	6.20E-08
*SFN*	0.17	0.0005	0.001	0.0008	0.0005	6.38E-08
*TPM3*	0.274	0.0005	0.0005	0.0005	0.0005	6.85E-08
*ZNF259*	5.00E-05	0.004	0.011	0.0075	0.1896	7.11E-08
*MAD2L2*	5.00E-05	0.0529	0.0713	0.0621	0.024	7.45E-08
*GSK3B*	5.00E-05	0.0969	0.2139	0.1554	0.01	7.77E-08
*SH3BP5*	0.003	0.0531	0.002	0.0276	0.001	8.27E-08
*CNDP2*	5.00E-05	0.0798	0.004	0.0419	0.0407	8.53E-08
*PRKD2*	5.00E-05	0.1108	0.1208	0.1158	0.015	8.69E-08
*CAPG*	0.142	0.0005	0.002	0.0013	0.0005	8.87E-08
*CAPNS1*	0.042	0.0005	0.008	0.0043	0.0005	8.93E-08
*YY1*	5.00E-05	0.2286	0.0988	0.1637	0.011	9.00E-08
*ACSL5*	5.00E-05	0.1375	0.0581	0.0978	0.019	9.29E-08
*CCT6A*	0.382	0.0005	0.0005	0.0005	0.0005	9.55E-08
*RPUSD3*	5.00E-05	0.1418	0.015	0.0784	0.025	9.80E-08
*SBF1*	0.006	0.008	0.0005	0.0043	0.004	1.02E-07
*YWHAE*	5.00E-05	0.031	0.024	0.0275	0.0739	1.02E-07
*XPO1*	0.274	0.0005	0.001	0.0008	0.0005	1.03E-07
*CRELD2*	5.00E-05	0.022	0.029	0.0255	0.0818	1.04E-07
*PDCD10*	5.00E-05	0.03	0.015	0.0225	0.0926	1.04E-07
*HNRPF*	5.00E-05	0.023	0.024	0.0235	0.0903	1.06E-07
*RFT1*	5.00E-05	0.03	0.005	0.0175	0.1231	1.08E-07
*BAX*	5.00E-05	0.3922	0.2326	0.3124	0.007	1.09E-07
*EFTUD2*	0.446	0.0005	0.0005	0.0005	0.0005	1.11E-07
*EEF1D*	0.448	0.0005	0.0005	0.0005	0.0005	1.12E-07
*FDPS*	0.032	0.001	0.013	0.007	0.0005	1.12E-07
***CDKN2A***	5.00E-05	0.012	0.0579	0.0349	0.0648	1.13E-07
*PFKP*	5.00E-05	0.03	0.001	0.0155	0.1476	1.14E-07
*TACC3*	5.00E-05	0.036	0.005	0.0205	0.117	1.20E-07
*FPGS*	0.0001	0.039	0.0659	0.0524	0.023	1.21E-07
*WDR74*	5.00E-05	0.1667	0.008	0.0873	0.028	1.22E-07
***CDKN2B***	5.00E-05	0.012	0.0667	0.0393	0.0632	1.24E-07
*SFPQ*	5.00E-05	1	0.0005	0.5002	0.005	1.25E-07
*NARS*	5.00E-05	0.4124	0.1465	0.2794	0.009	1.26E-07
*TCOF1*	5.00E-05	0.02	0.0475	0.0338	0.0756	1.28E-07
*CHAF1A*	0.0001	0.2581	0.0667	0.1624	0.008	1.30E-07
*ALDH18A1*	5.00E-05	0.2273	0.0629	0.1451	0.018	1.31E-07
*MGAT4B*	0.532	0.0005	0.0005	0.0005	0.0005	1.33E-07
*CYP2C9*	5.00E-05	0.7843	1	0.8922	0.003	1.34E-07
*MRPL37*	5.00E-05	0.0488	0.011	0.0299	0.0895	1.34E-07
*TTBK2*	5.00E-05	0.037	0.0879	0.0625	0.0438	1.37E-07
*AP3D1*	0.0008	0.026	0.001	0.0135	0.013	1.40E-07
*PDCD6IP*	5.00E-05	0.2597	0.039	0.1494	0.019	1.42E-07
*CLTA*	0.002	0.002	0.0693	0.0357	0.002	1.43E-07
*CCNI*	5.00E-05	0.03	0.0005	0.0152	0.1914	1.46E-07
*ZFYVE19*	5.00E-05	0.2516	0.0593	0.1555	0.019	1.48E-07

The top 154 recessive cancer genes have combined recessive cancer gene p-values lower than 1.5E-07. Alongside the Gene symbol, the p-values for each one of the 3 independent mutational events, i.e. amino acid substitution (pAA-NSSR), frameshift (pFrameshift), gene deletion (pDeletion) and the combined p-values are indicated. The pAA-NSSR p-value was first obtained as the average of pAA and pNSSR, two non independent p-values. The global recessive cancer gene p-value (pRecessiveCancer) was then calculated by multiplying the three independent p-values.

**Table 2:** The candidate recessive cancer genes with genomic location and associated copy number variations.

GENE SYMBOL	CHROMOSOMAL LOCATION	pRecessive Cancer	Gene Length	Copy Number Polymorphism
*NASP*	chr1:45822303-45857154	1.25E-11	34851	
*CCNB1*	chr5:68498668-68509822	2.50E-11	11154	
*DDX21*	chr10:70385897-70414285	1.00E-10	28388	
*DHX9*	chr1:181075073-181123505	1.00E-10	48432	
*GANAB*	chr11:62148878-62170680	1.00E-10	21802	
*ILF3*	chr19:10625987-10664093	1.25E-10	38106	
*AIPL1*	chr17:6267783-6279243	1.63E-10	11460	
*NOLC1*	chr10:103901922-103913617	1.75E-10	11695	
*MYO1C*	chr17:1314229-1335801	2.25E-10	21572	
*NUDC*	chr1:27120810-27145474	3.00E-10	24664	
*PGAM1*	chr10:99176016-99183187	5.00E-10	7171	
*IPO4*	chr14:23719265-23727964	5.25E-10	8699	
*XRCC5*	chr2:216682376-216779248	5.25E-10	96872	
*MTO1*	chr6:74228208-74267896	5.60E-10	39688	
*ANP32B*	chr9:99785309-99818043	6.02E-10	32734	
***TP53***	chr17:7512444-7531642	6.63E-10	19198	
*AFG3L2*	chr18:12319107-12367194	7.50E-10	48087	cnp1251
*FAF1*	chr1:50679522-51198524	9.96E-10	519002	
*CALR*	chr19:12910422-12916303	1.00E-09	5881	
*SREBF2*	chr22:40559051-40632319	1.00E-09	73268	
*XRCC6*	chr22:40347240-40389998	1.12E-09	42758	
*ARMC8*	chr3:139388837-139498909	1.25E-09	110072	cnp270
*GTPBP4*	chr10:1024348-1053704	1.40E-09	29356	
*HSPA4*	chr5:132415560-132468607	1.70E-09	53047	
*HDAC1*	chr1:32530294-32571811	1.82E-09	41517	
*PGD*	chr1:10381671-10402787	1.89E-09	21116	cnp10
*VCP*	chr9:35046560-35062564	2.00E-09	16004	
*ATXN2L*	chr16:28741914-28756057	2.50E-09	14143	cnp1177
*RPL6*	chr12:111327376-111331826	2.50E-09	4450	
*SARS*	chr1:109558062-109582308	2.56E-09	24246	
*NCL*	chr2:232027703-232037449	3.00E-09	9746	
*PTPRC*	chr1:196874759-196993168	3.00E-09	118409	
*SMARCA4*	chr19:10932605-11033952	3.00E-09	101347	
*CCT3*	chr1:154545375-154574819	3.20E-09	29444	
*NET1*	chr10:5478545-5490424	3.58E-09	11879	
*HNRPD*	chr4:83493490-83514173	3.74E-09	20683	
*SQSTM1*	chr5:179180502-179197681	3.75E-09	17179	
*TUBB2C*	chr9:139255531-139257980	3.75E-09	2449	
*C1QBP*	chr17:5276822-5283195	4.00E-09	6373	
*TRAP1*	chr16:3648038-3707599	4.00E-09	59561	
*ALDOA*	chr16:29984544-29989235	4.50E-09	4691	cnp1179
*RNASEH2A*	chr19:12778427-12785462	4.59E-09	7035	
*DDX24*	chr14:93587021-93617311	5.00E-09	30290	
*ILVBL*	chr19:15086786-15097577	5.00E-09	10791	cnp1283
*SERPINB3*	chr18:59473411-59480094	5.05E-09	6683	
*UQCRC1*	chr3:48611435-48622102	6.60E-09	10667	
*EEF2*	chr19:3927054-3936461	7.00E-09	9407	
*NUSAP1*	chr15:39412360-39460537	7.43E-09	48177	
*DNAJC11*	chr1:6616817-6684460	8.66E-09	67643	
*HSP90AA1*	chr14:101616827-101675839	9.00E-09	59012	
*MYH9*	chr22:35007271-35113927	9.12E-09	106656	
*HK1*	chr10:70748628-70831641	9.35E-09	83013	
*IARS*	chr9:94012445-94095859	1.00E-08	83414	
*YBX1*	chr1:42920652-42940604	1.00E-08	19952	
*HDLBP*	chr2:241815351-241903927	1.12E-08	88576	
*EWSR1*	chr22:27994016-28026515	1.25E-08	32499	
*DHX15*	chr4:24138187-24195282	1.27E-08	57095	
*SERPINB4*	chr18:59455474-59462482	1.28E-08	7008	
*POLR2A*	chr17:7328421-7358653	1.29E-08	30232	
*ALG14*	chr1:95220884-95311071	1.50E-08	90187	
*PRMT1*	chr19:54872307-54883516	1.50E-08	11209	
*COX4NB*	chr16:84369736-84390601	1.57E-08	20865	
*SPTBN1*	chr2:54536957-54752086	1.58E-08	215129	
*PTPRF*	chr1:43769133-43861929	1.61E-08	92796	
*KHDRBS1*	chr1:32252077-32282058	1.62E-08	29981	
*PABPC1*	chr8:101784319-101803491	1.75E-08	19172	
*CTNNA1*	chr5:138117005-138298621	1.80E-08	181616	
*DDB1*	chr11:60823494-60857242	1.80E-08	33748	cnp921
*GNB2L1*	chr5:180596533-180603512	1.85E-08	6979	
*WDR1*	chr4:9685060-9727671	2.00E-08	42611	cnp312
*AARS*	chr16:68843797-68880913	2.10E-08	37116	cnp1189
*NDE1*	chr16:15651604-15726490	2.10E-08	74886	
*NQO1*	chr16:68300805-68318034	2.25E-08	17229	
*RUVBL2*	chr19:54188967-54210994	2.30E-08	22027	
*ZWINT*	chr10:57787204-57791040	2.37E-08	3836	
*HP1BP3*	chr1:20941757-20985768	2.52E-08	44011	
*WDR79*	chr17:7532519-7547544	2.60E-08	15025	
*SLC25A6*	chrY:1465044-1470998	2.75E-08	5954	
*TYMS*	chr18:647650-663492	2.76E-08	15842	
*SLC25A3*	chr12:97511533-97519908	3.00E-08	8375	
*ACLY*	chr17:37276706-37328798	3.49E-08	52092	
*ALDH3A1*	chr17:19581891-19592200	3.50E-08	10309	
*TTC8*	chr14:88360730-88414087	3.55E-08	53357	
*YME1L1*	chr10:27439390-27483327	3.59E-08	43937	
*ATP5A1*	chr18:41918107-41938197	3.61E-08	20090	
*MRPS2*	chr9:137532374-137536337	3.69E-08	3963	
*HNRPH3*	chr10:69761884-69772952	3.70E-08	11068	
*IMMT*	chr2:86224565-86276404	4.20E-08	51839	
*IMPDH2*	chr3:49036771-49041879	4.35E-08	5108	
*NCKAP1*	chr2:183497850-183611474	4.36E-08	113624	cnp194
*TTLL12*	chr22:41892572-41913051	4.39E-08	20479	
***PTEN***	chr10:89613174-89718511	4.45E-08	105337	
*WBSCR16*	chr7:74094219-74127635	4.55E-08	33416	cnp627
*XPNPEP1*	chr10:111614513-111673192	4.65E-08	58679	
*SREBF1*	chr17:17656110-17681050	4.78E-08	24940	
*CCDC5*	chr18:41938322-41962296	5.02E-08	23974	
*DDX19B*	chr16:68890572-68925230	5.03E-08	34658	
*MAPK6*	chr15:50098738-50145751	5.21E-08	47013	
*MAP4*	chr3:47867189-48105715	5.24E-08	238526	
*PHB2*	chr12:6944777-6950152	5.50E-08	5375	
*SAE1*	chr19:52325983-52405371	5.60E-08	79388	
*TALDO1*	chr11:737431-755023	5.73E-08	17592	cnp884
*AHCY*	chr20:32331736-32354784	5.75E-08	23048	
*GTF3C1*	chr16:27379435-27468752	5.82E-08	89317	
*PRPF19*	chr11:60414782-60430632	5.99E-08	15850	
*LASP1*	chr17:34279893-34331540	6.06E-08	51647	
*TRIP10*	chr19:6690706-6702528	6.07E-08	11822	
*HSPD1*	chr2:198059554-198073243	6.10E-08	13689	
*EIF4G2*	chr11:10775169-10787158	6.20E-08	11989	
*SFN*	chr1:27062219-27063534	6.38E-08	1315	
*TPM3*	chr1:152400913-152431233	6.85E-08	30320	
*ZNF259*	chr11:116154486-116163949	7.11E-08	9463	
*MAD2L2*	chr1:11657124-11663774	7.45E-08	6650	
*GSK3B*	chr3:121028237-121295203	7.77E-08	266966	
*SH3BP5*	chr3:15271360-15357905	8.27E-08	86545	
*CNDP2*	chr18:70314576-70339336	8.53E-08	24760	
*PRKD2*	chr19:51869412-51912224	8.69E-08	42812	
*CAPG*	chr2:85475381-85491187	8.87E-08	15806	
*CAPNS1*	chr19:41322757-41333094	8.93E-08	10337	
*YY1*	chr14:99774854-99814557	9.00E-08	39703	
*ACSL5*	chr10:114125945-114178127	9.29E-08	52182	
*CCT6A*	chr7:56086871-56099176	9.55E-08	12305	
*RPUSD3*	chr3:9854533-9860676	9.80E-08	6143	
*SBF1*	chr22:49232101-49260320	1.02E-07	28219	
*YWHAE*	chr17:1194594-1250267	1.02E-07	55673	
*XPO1*	chr2:61558573-61618922	1.03E-07	60349	
*CRELD2*	chr22:48698347-48707178	1.04E-07	8831	
*PDCD10*	chr3:168884389-168935345	1.04E-07	50956	
*HNRPF*	chr10:43201070-43223305	1.06E-07	22235	
*RFT1*	chr3:53099850-53139503	1.08E-07	39653	
*BAX*	chr19:54149928-54156867	1.09E-07	6939	
*EFTUD2*	chr17:40283804-40332289	1.11E-07	48485	
*EEF1D*	chr8:144733040-144750726	1.12E-07	17686	
*FDPS*	chr1:153546200-153557080	1.12E-07	10880	cnp61
***CDKN2A***	chr9:21957751-21984490	1.13E-07	26739	
*PFKP*	chr10:3099751-3168995	1.14E-07	69244	cnp816
*TACC3*	chr4:1693063-1716693	1.20E-07	23630	cnp308
*FPGS*	chr9:129605328-129616377	1.21E-07	11049	
*WDR74*	chr11:62356959-62364204	1.22E-07	7245	
***CDKN2B***	chr9:21992905-21999312	1.24E-07	6407	
*SFPQ*	chr1:35421789-35431322	1.25E-07	9533	
*NARS*	chr18:53418891-53440175	1.26E-07	21284	
*TCOF1*	chr5:149717427-149760063	1.28E-07	42636	
*CHAF1A*	chr19:4353659-4394393	1.30E-07	40734	
*ALDH18A1*	chr10:97355676-97406557	1.31E-07	50881	
*MGAT4B*	chr5:179156710-179166547	1.33E-07	9837	
*CYP2C9*	chr10:96688429-96739137	1.34E-07	50708	
*MRPL37*	chr1:54438427-54456638	1.34E-07	18211	
*TTBK2*	chr15:40823837-41000299	1.37E-07	176462	
*AP3D1*	chr19:2051993-2102556	1.40E-07	50563	
*PDCD6IP*	chr3:33814560-33886198	1.42E-07	71638	
*CLTA*	chr9:36180891-36202055	1.43E-07	21164	
*CCNI*	chr4:78188198-78216149	1.46E-07	27951	
*ZFYVE19*	chr15:38886565-38894059	1.48E-07	7494	

The top 154 genes have combined recessive cancer gene p-values lower than 1.5×10^−7^ (FDR = 0.39). Alongside the gene symbol, genome coordinates, gene length, cancer gene p-value and eventual copy number polymorphic site are reported.

The probability of a protein having amino acid mutations and frame-shifts in cancer, events which are independent, was defined as the product of the respective p-values. Just using these two tests, the prototypical *TP53* and *PTEN* cancer genes ranked 205^th^ and 233^rd^ out of 27,184 evaluated human transcripts (p-value<1×10^−4^). Additionally, two other well-known recessive cancer genes, *CDKN2A* and *CDKN2B*, also had significant p-values, albeit lower rankings (p<0.0025 and FDR = 0.019, respectively). This behavior was expected for genes with small coding regions, which might be more commonly deleted than mutated [6]. Their presence in the significant point mutations cancer gene-set, even at this intermediate stage, reassured us of the selection capabilities of our algorithm. Nevertheless this early classification, based entirely on point mutations, was compiled only from two mutation tests; thus, relying on EST sequencing data, it was still not reliable according to our model which incorporated an additional mutation mode. It should be noted that we did not set to identify translocations, alterations expected to be dominant at the cellular level and therefore not suited to our quest for recessive genes.

The last test, based on aCGH analysis, confirmed that a very large portion of the human genome is frequently deleted in cancer. As expected for our 2-channels aCGH procedure, we correctly detected sex chromosome genes as differentially represented in the genome screens. In particular, owing to the resolution of our structural assay, the genes from the pseudo-autosomal region 1 were identified as normal diploid (supplemental Figure S1). Most importantly, we would expect that polymorphic CNVs had not filtered through the aCGH assay. Indeed, only a small percentage of cancer genes coincided with polymorphic CNVs and this percentage is even smaller than expected by chance (Table 2).

The number of deletions detected by aCGH in the cancer genome is very high (more than 10% of human genes were deleted in cancer). Notwithstanding this deletion excess, when all mutation modes are included, the number of candidate genes is less than 0.5% of the analyzed human genome.

The cancer gene products are involved in biological processes such as cell cycle, DNA repair and apoptosis, in agreement with literature. The same functional terms are also associated to the genes in the COSMIC Cancer Census [18]. Strikingly, tyrosine kinases, dominant oncogenes, present in the Cancer Census, were absent from our cancer gene-set, in agreement with the selection for recessive genes.

Some strong limitations are inherent to our approach. It is unlikely that the recorded frame-shifts are polymorphisms, since they alter the primary structure of the gene products. Conversely, they might be very often results of sequencing errors. For this reason, we chose to filter out as much as possible the sequencing errors by using a paired t test over a sliding window. Another controversy might be related to the somatic character of the detected mutations. Since there are virtually no germ-line sequences corresponding to the tumor libraries in the EST database, there can not be any formal demonstration that the selected genes correspond to somatic mutation targets. We can not establish how many of the detected mismatches are real mutations, nor how many of them are truly of somatic origin. We could only attach to each human gene a p-value for the excess of mismatches with gene inactivating potential in cancer samples. The presence of *TP53*, *PTEN* and *CDKN2A* in the candidate gene-set and its functional characteristics, are evidences in favor of the hypothesis that we measured an excess of somatic cancer mutations. We will be able to refute this hypothesis by using various experimental protocols. On the other hand, it is possible that some of the candidate genes might bear germ-line mutations and thus constitute predisposition traits for cancer insurgence.

When we compared our results to those of the recently published massive sequencing project, some differences emerged. We used a larger amount of sequencing data, albeit of lower quality since we did not use second pass sequencing data. We obtained from dbEST a number of mismatches roughly 5 times higher than the genome wide sequencing screens. This excess could be due to the lower quality sequencing data in ESTs or the higher sensitivity of our approach compared to PCR based direct sequencing. Detection of under-represented mutations in often heterogeneous cancer biopsies can be a technical challenge for direct sequencing, but not for cloned ESTs.

ESTs were used in previous attempts to identify cancer related genes. Almost invariably these approaches were based on expression profiling, which in tumor samples is probably correlates and late events, among the steps leading to tumor development and progression. In a very different data mining effort on EST sequences in cancer, Qiu and co-workers [20] measured SNP-tumor association. Their analysis was highly focused on single nucleotide mismatches, and restricted to known mutations described in the SNP database and present in at least 50 EST hits. They identified 4,865 SNP frequent in tumors (p<0.05), out of which 327 induced amino acid substitution (cSNP). Many major histocompatibility complex (MHC) class II molecules were present among these coding SNPs, while none was present in our recessive cancer gene-set. Most importantly, no landmark cancer genes, such as *TP53*, *PTEN* and *CDKN2A* were present within cSNPs. Finally, none of the SNP genes detected by Qiu et al. [20] were present in our candidate recessive cancer gene set.

The minute cancer recessive sub-genome (<0.5%) we identified might represent a milestone towards the identification of novel markers for early diagnosis and prognosis. Additionally, our mining strategy can be applied to the data which will be available upon the sequencing of cancer genomes [22]. Finally, our work might lead to a different equilibrium within the pool of cancer genes, currently unbalanced towards dominant oncogenes.

## Materials and Methods

### EST data mining

All human coding sequences were extracted from RefSeq mRNA database at NCBI (27,184 sequences). The dbEST database contained more than 5.6 million human ESTs (exceeding 3,009 million nucleotides in length). The dbEST libraries (7574) were manually annotated corresponding to the biomaterial of origin and ESTs were subdivided in the following seven classes: cancer tissues and cell lines (Y, 4466 libraries), normal tissues (N, 2621), cell lines of uncertain origin (C, 193), hyperplasia (B, 32), normal tissues associated to cancer lesions (A, 33), matched normal controls from cancer patients (M, 70) and undetermined origin (U, 159). Only the library with clear cut origin was used: i.e. 4466 cancer tissues and cell lines (Y) vs. 2621 normal tissues (N). Tissues associated (A) or matched (M) to cancer, benign tumors (B) and other cell lines (C) were not used. The coding sequences for each RefSeq entry were aligned against the human dbEST database by using BLAST. The Cancer Mutome MySQL database was populated with a total of 43,965,904 mismatches and gaps extracted from 3,839,543 alignments. Perl was used to develop all the scripts and implement the system. BioPerl was used for the BLAST procedure and parsing. BLAST parameters were set to default (expect = 1E-21) with the exception of recovering up to a maximum of 500 alignments for each query.

### Detection of point mutations in ESTs

To attenuate the problem of high sequencing error rate in ESTs, our procedure retrieved candidate mutations only in the region of maximum nucleotide identity to the query. Our assumption was that an identical error rate was present in the two EST populations, those derived from the control and those from the cancer cells. Therefore the frequencies of mismatches due to sequencing errors are expected to be comparable across ESTs for the same genes. The mismatches were considered for subsequent analysis only when present in the internal sequence (not in the first or last ten nucleotides of the BLAST alignments). Mismatches were then evaluated for their capabilities of changing the amino acid residue in the correspondent codon. A single candidate mutation was considered only once for each dbEST library, to avoid bias due to RNA copy number. The 8972 human genes most variable for number of mismatches (IQR>0.5) were retained for further testing. Statistics for amino acid substitutions, non-synonymous to synonymous nucleotide exchange rate and frame-shifts were calculated for each human coding sequence. Gene specific confidence limits for the respective paired t tests were calculated by bootstrap analysis. The two bootstrap classes were composed by random extracting 1000 times, with replacement, cancer or normal status from the library classes [23], [24].

In the first of three different measures, the frequencies of amino acid substitution were compared, for each gene in normal and cancerous tissues, by using paired t test over a 25-residues protein window. Normalization of mismatches for the control and cancer classes was attained by using a gene specific and local correction factor. The correction factor was derived by dividing the respective counts of ESTs in both classes at each nucleotide position of the query. The score assigned to each human RefSeq gene corresponded to the sum of the T scores values exceeding the gene-specific confidence limit (p<0.05) over the sliding window (i.e. the area of the peaks above the threshold).

The second measure, linked to the amino acid substitution frequency consisted in the evaluation of the selective pressure for amino acids changes. This filter was implemented to separate causal from bystander mutations and to further diminish the effects of sequencing errors. The ratios of non-synonymous (NS) to synonymous (S) nucleotide substitutions within the cancer and normal ESTs were calculated for each gene. A paired t test was used to compare the cancer and normal NS/S substitution ratios at different codons. When the number of synonymous substitutions at denominator was null, unity was added to both numerator and denominator. Only the amino acid positions significant for frequency of substitutions (in the amino acid substitution test above) were evaluated here. The gene specific confidence limit at 5%, the p-values and the FDR were again computed by bootstrap, as described above.

A third measure on point mutations was relative to the frequency of frame-shifts, which can produce premature protein termination or other major alterations in primary structure. In a paired t test, analogous to that for the pAA, a 25-nt sliding window based procedure was applied to the number of frame-shifts induced in cancer by 1 nucleotide insertions or deletions. Longer DNA alterations were not recorded, and were extremely rare. The gene p-values for such frame-shifts in cancer were again computed by bootstrap and defined as pFrameshift.

### ESTs P-value and false detection rate calculation

Procedures were devised for calculation of gene-specific p-values and false detection rates in each one of the described approaches. Bootstrap analysis was used to compute the adjusted probability that a human gene was affected in cancer but not in normal ESTs [23], [24]. The resampling test allowed us to define confidence limits for each different gene and to effectively tackle local issues such as DNA composition, CpG occurrence, and protein or gene length. For the point mutation analyses, the resampling procedure was performed only on the protein residues found to be above T threshold (p<0.05). A range of bootstraps were performed to choose the lowest number of resampling cycles yielding stable p-values through a short gene list and 1000 cycles were found to be a satisfactory requirement. The ESTs belonging to cancer and normal classes were randomly subdivided to form two simulated classes with the same size as the original ones. The gene specific p-value was defined as the frequency at which the resampling test scored equal or better than the real test. Null p-values were set to half of the lowest p-value in the respective simulations.

### Detection of deletions in array CGH

744 comparative genomic hybridization arrays were studied (537 samples from GEO and 207 from SMD). All platforms were 2-channel based, data were downloaded as normalized values, and probes were indexed by gene symbol. Gene data and annotations were stored in the GeoSoft database. All normalized log ratios were converted to log2 ratios, with the cancer value at the numerator and the control value at the denominator. Pre-filtering of genes was performed on standard deviation, to exclude the genes which did not show high variation of their genomic profiles (std dev<0.2). Genes were scored when measured in at least 300 tumors. Deleted cancer genes were expected to have log_2_ ratios lower than the 5^th^ percentile of the bootstrapped log_2_ ratios; amplified genes log_2_ ratios higher than the 95^th^ percentile of the bootstrapped values. Bootstrap analysis was used (10,000 random swaps of tumor and control channels) to obtain gene specific p-values and confidence limits for deletion and amplification.

### Point Mutation and aCGH combined p-values

Finally, the p-values obtained by the three different tests: pAA-NSSR, pFrameshift and pDeletion were multiplied together to compute the global pRecessiveCancer p-value. This p-value was used to sort the human genes by their propensity to bear mutations in cancer. One hundred and 54 genes were selected with p-value below 1.5×10^−7^. One hundred resampling cycles were performed by randomly associating p-values for each mutation test and yielded a false detection rate of 39%. EASE (http://david.abcc.ncifcrf.gov) and BinGO (Cytoscape plugin) were used for Gene Ontology analysis. Hyper-geometric test with Benjamini and Hochberg false discovery rate correction was used in BinGO [25].

## Supporting Information

Figure S1 Genomic structures are correctly identified by the aCGH protocol. Track analysis in UCSC Genome Browser of Xp22 Pseudo-Autosomal Region 1 (PAR1). The Pseudo-Autosomal Region 1 is correctly identified as normal (diploid) by the array CGH analysis, while the rest of X chromosome is reported, also as expected, “pseudo-amplified”. The chromosome X genes 3 prime of PAR1 appear as amplified because their DNA copy number is higher than expected when compared to the respective average DNA copy number in the whole, mixed sex, tumour population.
(0.31 MB TIF)

Figure S2 Distribution of gene size in the candidate recessive cancer gene-set. The recessive cancer gene sizes do not differ significantly from the gene sizes in the human genome (most common genes range between 32 and 128 kb).
(0.28 MB TIF)

Table S1 Array CGH datasets.
(0.04 MB DOC)

Table S2 Functional (Gene ontology, biological process) chart of the candidate cancer recessive genes, FDR<0.5
(0.22 MB DOC)

## References

[1] Croce CM (2008). Oncogenes and cancer.. N Engl J Med.

[2] Reddy EP, Reynolds RK, Santos E, Barbacid M (1982). A point mutation is responsible for the acquisition of transforming properties by the T24 human bladder carcinoma oncogene.. Nature.

[3] Tabin CJ, Bradley SM, Bargmann CI, Weinberg RA, Papageorge AG (1982). Mechanism of activation of a human oncogene.. Nature.

[4] Douma S, Van Laar T, Zevenhoven J, Meuwissen R, Van Garderen E (2004). Suppression of anoikis and induction of metastasis by the neurotrophic receptor TrkB.. Nature.

[5] Schlabach MR, Luo J, Solimini NL, Hu G, Xu Q (2008). Cancer proliferation gene discovery through functional genomics.. Science.

[6] Murphy JA, Barrantes-Reynolds R, Kocherlakota R, Bond JP, Greenblatt MS (2004). The CDKN2A database: Integrating allelic variants with evolution, structure, function, and disease association.. Hum Mutat.

[7] Olivier M, Eeles R, Hollstein M, Khan MA, Harris CC (2002). The IARC TP53 database: new online mutation analysis and recommendations to users.. Hum Mutat.

[8] Stenson PD, Ball EV, Mort M, Phillips AD, Shiel JA (2003). Human Gene Mutation Database (HGMD): 2003 update.. Hum Mutat.

[9] Garnett MJ, Marais R (2004). Guilty as charged: B-RAF is a human oncogene.. Cancer Cell.

[10] Wang Z, Shen D, Parsons DW, Bardelli A, Sager J (2004). Mutational analysis of the tyrosine phosphatome in colorectal cancers.. Science.

[11] (1997). (1997) Pathology of familial breast cancer: differences between breast cancers in carriers of BRCA1 or BRCA2 mutations and sporadic cases. Breast Cancer Linkage Consortium.. Lancet.

[12] Friend SH, Bernards R, Rogelj S, Weinberg RA, Rapaport JM (1986). A human DNA segment with properties of the gene that predisposes to retinoblastoma and osteosarcoma.. Nature.

[13] Greenman C, Stephens P, Smith R, Dalgliesh GL, Hunter C (2007). Patterns of somatic mutation in human cancer genomes.. Nature.

[14] Kantarjian H, Sawyers C, Hochhaus A, Guilhot F, Schiffer C (2002). Hematologic and cytogenetic responses to imatinib mesylate in chronic myelogenous leukemia.. N Engl J Med.

[15] Davies H, Bignell GR, Cox C, Stephens P, Edkins S (2002). Mutations of the BRAF gene in human cancer.. Nature.

[16] Wood LD, Parsons DW, Jones S, Lin J, Sjoblom T (2007). The genomic landscapes of human breast and colorectal cancers.. Science.

[17] Samuels Y, Wang Z, Bardelli A, Silliman N, Ptak J (2004). High frequency of mutations of the PIK3CA gene in human cancers.. Science.

[18] Futreal PA, Coin L, Marshall M, Down T, Hubbard T (2004). A census of human cancer genes.. Nat Rev Cancer.

[19] Irizarry K, Kustanovich V, Li C, Brown N, Nelson S (2000). Genome-wide analysis of single-nucleotide polymorphisms in human expressed sequences.. Nat Genet.

[20] Qiu P, Wang L, Kostich M, Ding W, Simon JS (2004). Genome wide in silico SNP-tumor association analysis.. BMC Cancer.

[21] Altschul SF, Gish W, Miller W, Myers EW, Lipman DJ (1990). Basic local alignment search tool.. J Mol Biol.

[22] Stratton M (2008). Genome resequencing and genetic variation.. Nat Biotechnol.

[23] Davison AC, Hinkley DV (1997). Bootstrap Methods and Their Application..

[24] Efron B, Tibshirani RJ (1993). An Introduction to the Bootstrap..

[25] Maere S, Heymans K, Kuiper M (2005). BiNGO: a Cytoscape plugin to assess overrepresentation of gene ontology categories in biological networks.. Bioinformatics.

