# Supplementary figures and images for: Genome Wide Identification of Recessive Cancer Genes by Combinatorial Mutation Analysis
Source: PLoS One. 2008 Oct 10;3(10):e3380. doi: 10.1371/journal.pone.0003380 (PMC2557123; doi:10.1371/journal.pone.0003380)

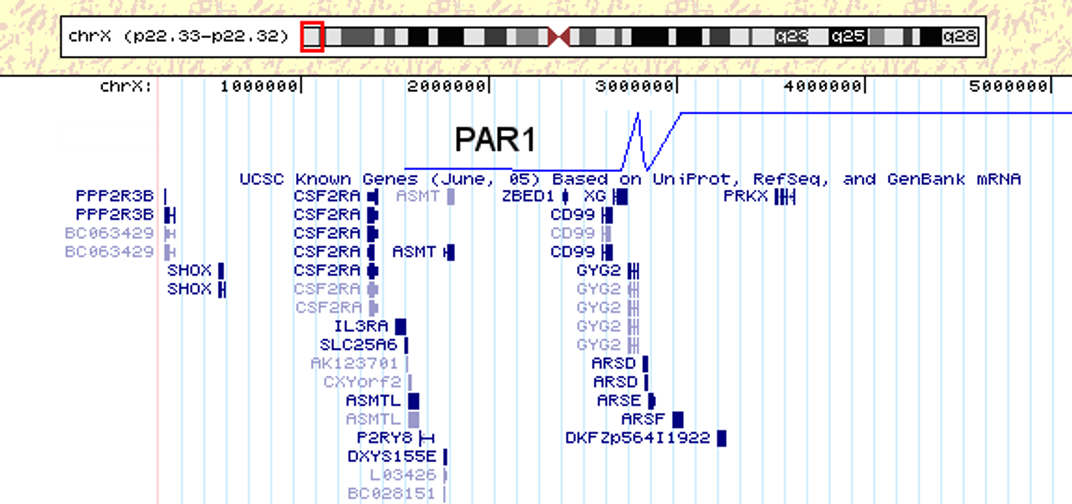

Supplement: Figure S1 — Genomic structures are correctly identified by the aCGH protocol. Track analysis in UCSC Genome Browser of Xp22 Pseudo-Autosomal Region 1 (PAR1). The Pseudo-Autosomal Region 1 is correctly identified as normal (diploid) by the array CGH analysis, while the rest of X chromosome is reported, also as expected, “pseudo-amplified”. The chromosome X genes 3 prime of PAR1 appear as amplified because their DNA copy number is higher than expected when compared to the respective average DNA copy number in the whole, mixed sex, tumour population. (0.31 MB TIF) [file pone.0003380.s001.tif]

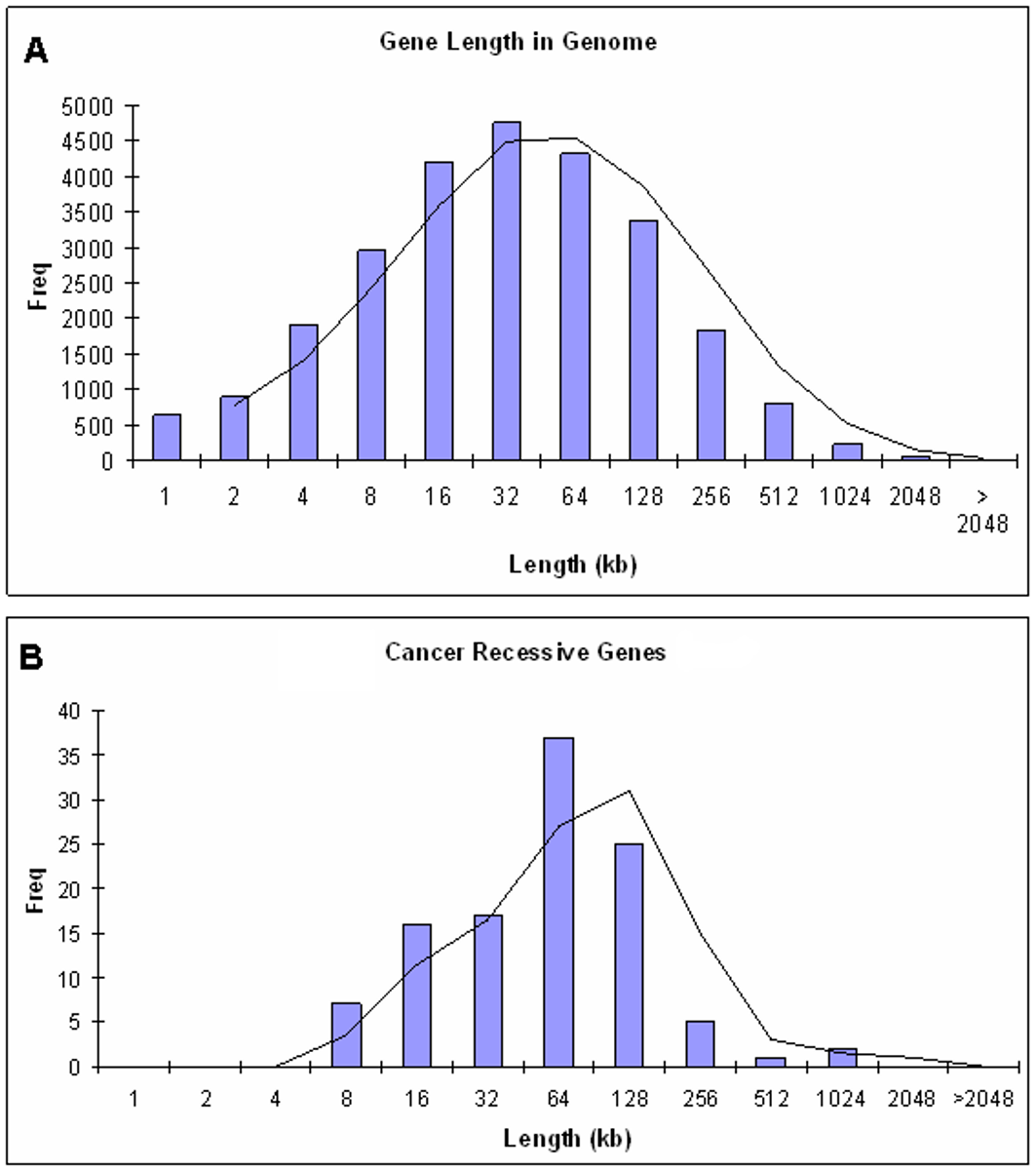

Supplement: Figure S2 — Distribution of gene size in the candidate recessive cancer gene-set. The recessive cancer gene sizes do not differ significantly from the gene sizes in the human genome (most common genes range between 32 and 128 kb). (0.28 MB TIF) [file pone.0003380.s002.tif]
