# Supplementary material for: Genome Wide Identification of Recessive Cancer Genes by Combinatorial Mutation Analysis
Source: PLoS One. 2008 Oct 10;3(10):e3380. doi: 10.1371/journal.pone.0003380 (PMC2557123; doi:10.1371/journal.pone.0003380)
Supplement: Table S1 — Array CGH datasets. (0.04 MB DOC) [file pone.0003380.s003.doc]

**Genome Wide Identification of Recessive Cancer Genes by Combinatorial Mutation Analysis.**

**Stefano Volinia^1,2^, Nicoletta Mascellani^1^, Jlenia Marchesini^1^, Angelo Veronese^3^, Elizabeth Ormondroyd^4^, Hansjuerg Alder^2^, Jeff Palatini^2^, Massimo Negrini^3^, Carlo M. Croce^2*^**

**Table S1. Array CGH datasets**

| **GEO datasets** | **# of**  **samples** | **Cancer type** | **Platform** |
| --- | --- | --- | --- |
| GSE4659 | 32 | AML | GPL2873 |
| GSE6472 | 6 | Nasopharyngeal carcinoma | GPL2879 |
| GSE7077 | 4 | Osteosarcoma | GPL2879 |
| GSE7344 | 16 | Glioma | GPL2873 |
| GSE7482 | 25 | ACC | GPL2879 |
| GSE7615 | 298 | Pancreas, glioblastoma, T-ALL, melanoma, colon | GPL2879  GPL4091 |
| GSE7822 | 14 | Melanoma | GPL2879 |
| GSE8398 | 25 | Ewing Sarcoma | GPL2879 |
| GSE8804 | 13 | Myelodysplasia | GPL2879 |
| GSE8918 | 87 | Follicular Lymphoma, CLL, Mantle Cell Lymphoma, Nodal Marginal Zone Lymphoma, Lymphoplasmacytic Lymphoma, Splenic Marginal Zone Lymphoma , MALT | GPL2879 |
| GSE9015 | 7 | Breast | GPL4091 |
| GSE9654 | 10 | Osteosarcoma | GPL2879 |
| SMD | 207 | Lung, Pancreas, Breast, Melanoma, Fibrous Histiocytoma | Stanford |

- GPL2873 Agilent- Human Genome CGH Microarray 44A G4410A
- GPL2879 Agilent- Human Genome CGH Microarray 44B G4410B
- GPL4091 Agilent- Human Genome CGH Microarray 244A G4411B
