# Supplementary material for: Genome Wide Identification of Recessive Cancer Genes by Combinatorial Mutation Analysis
Source: PLoS One. 2008 Oct 10;3(10):e3380. doi: 10.1371/journal.pone.0003380 (PMC2557123; doi:10.1371/journal.pone.0003380)
Supplement: Table S2 — Functional (Gene ontology, biological process) chart of the candidate cancer recessive genes, FDR<0.5 (0.22 MB DOC) [file pone.0003380.s004.doc]

**Genome Wide Identification of Recessive Cancer Genes by Combinatorial Mutation Analysis.**

**Stefano Volinia^1,2^, Nicoletta Mascellani^1^, Jlenia Marchesini^1^, Angelo Veronese^3^, Elizabeth Ormondroyd^4^, Hansjuerg Alder^2^, Jeff Palatini^2^, Massimo Negrini^3^, Carlo M. Croce^2*^**

**Table S2.** Functional (Gene ontology, biological process) chart of the candidate cancer recessive genes, FDR <0.5

| **Term** | **Count** | **%** | **p-value** | **Genes** |
| --- | --- | --- | --- | --- |
| GO:0007049~cell cycle | 25 | 16.34% | 1.120E-06 | *MAD2L2, CCNI, NOLC1, PTEN, NUDC, KHDRBS1, NDE1, NASP, ZWINT, CALR, DDB1, SFN, GTPBP4, NUSAP1, PTPRC, CCNB1, CCDC5, CDKN2B, CDKN2A, TP53, EIF4G2, CHAF1A, ILF3, MAPK6, BAX,* |
| GO:0022402~cell cycle process | 22 | 14.38% | 2.920E-06 | *MAD2L2, CCNI, CCDC5, NOLC1, KHDRBS1, NUDC, PTEN, CDKN2A, CDKN2B, TP53, NDE1, ZWINT, CALR, EIF4G2, DDB1, GTPBP4, SFN, ILF3, PTPRC, NUSAP1, BAX, CCNB1,* |
| GO:0022403~cell cycle phase | 15 | 9.80% | 3.600E-06 | *MAD2L2, CCDC5, NOLC1, NUDC, KHDRBS1, CDKN2B, CDKN2A, TP53, NDE1, ZWINT, CALR, ILF3, PTPRC, NUSAP1, CCNB1,* |
| GO:0000074~regulation of progression through cell cycle | 18 | 11.76% | 4.450E-06 | *MAD2L2, CCNI, KHDRBS1, NUDC, PTEN, CDKN2A, CDKN2B, TP53, ZWINT, CALR, EIF4G2, DDB1, GTPBP4, SFN, PTPRC, NUSAP1, BAX, CCNB1,* |
| GO:0051726~regulation of cell cycle | 18 | 11.76% | 4.800E-06 | *MAD2L2, CCNI, KHDRBS1, NUDC, PTEN, CDKN2A, CDKN2B, TP53, ZWINT, CALR, EIF4G2, DDB1, GTPBP4, SFN, PTPRC, NUSAP1, BAX, CCNB1,* |
| GO:0006396~RNA processing | 16 | 10.46% | 1.410E-05 | *NOLC1, HNRPF, KHDRBS1, CDKN2A, DHX15, SFPQ, SARS, RPUSD3, HNRPH3, HNRPD, YBX1, MTO1, PABPC1, PRPF19, AARS, EFTUD2,* |
| GO:0009057~macromolecule catabolic process | 15 | 9.80% | 4.190E-05 | *CDKN2A, HNRPD, PFKP, ALDOA, HK1, RNASEH2A, SQSTM1, AFG3L2, PGD, YME1L1, BAX, PGAM1, FAF1, TALDO1, VCP,* |
| GO:0006996~organelle organization and biogenesis | 26 | 16.99% | 5.000E-05 | *NOLC1, NDE1, NASP, ZWINT, MAP4, CALR, CAPG, HSPD1, SFN, SLC25A6, GTPBP4, SMARCA4, RUVBL2, MYH9, NUSAP1, TUBB2C, TRIP10, HSP90AA1, CDKN2A, TP53, HP1BP3, HDAC1, CHAF1A, SPTBN1, BAX, LASP1,* |
| GO:0000279~M phase | 12 | 7.84% | 5.270E-05 | *MAD2L2, CCDC5, NOLC1, NUDC, CDKN2B, TP53, ILF3, NDE1, ZWINT, NUSAP1, CALR, CCNB1,* |
| GO:0000278~mitotic cell cycle | 12 | 7.84% | 1.310E-04 | *MAD2L2, CCDC5, NOLC1, KHDRBS1, NUDC, CDKN2A, CDKN2B, TP53, NDE1, ZWINT, NUSAP1, CCNB1,* |
| GO:0016265~death | 20 | 13.07% | 1.520E-04 | *PDCD6IP, PTEN, CDKN2A, TP53, CALR, EIF4G2, HDAC1, HSPD1, SLC25A6, SFN, PDCD10, NCKAP1, CTNNA1, SQSTM1, PTPRC, FAF1, GSK3B, BAX, TUBB2C, VCP,* |
| GO:0008219~cell death | 20 | 13.07% | 1.520E-04 | *PDCD6IP, PTEN, CDKN2A, TP53, CALR, EIF4G2, HDAC1, HSPD1, SLC25A6, SFN, PDCD10, NCKAP1, CTNNA1, SQSTM1, PTPRC, FAF1, GSK3B, BAX, TUBB2C, VCP,* |
| GO:0006974~response to DNA damage stimulus | 12 | 7.84% | 1.550E-04 | *SFN, TYMS, TP53, PRPF19, RUVBL2, SFPQ, BAX, XRCC6, XRCC5, VCP, CHAF1A, DDB1,* |
| GO:0007005~mitochondrion organization and biogenesis | 7 | 4.58% | 1.720E-04 | *HSPD1, SFN, SLC25A6, CDKN2A, HSP90AA1, TP53, BAX,* |
| GO:0006457~protein folding | 11 | 7.19% | 1.840E-04 | *HSPD1, HSPA4, CCT6A, HSP90AA1, RUVBL2, AIPL1, CALR, TRAP1, CCT3, CHAF1A, DNAJC11,* |
| GO:0007067~mitosis | 10 | 6.54% | 2.000E-04 | *MAD2L2, CCDC5, NOLC1, NUDC, CDKN2B, TP53, NDE1, ZWINT, NUSAP1, CCNB1,* |
| GO:0006915~apoptosis | 19 | 12.42% | 2.090E-04 | *PDCD6IP, PTEN, CDKN2A, TP53, CALR, HDAC1, HSPD1, SLC25A6, SFN, PDCD10, NCKAP1, SQSTM1, CTNNA1, PTPRC, FAF1, GSK3B, BAX, TUBB2C, VCP,* |
| GO:0000087~M phase of mitotic cell cycle | 10 | 6.54% | 2.130E-04 | *MAD2L2, CCDC5, NOLC1, NUDC, CDKN2B, TP53, NDE1, ZWINT, NUSAP1, CCNB1,* |
| GO:0000075~cell cycle checkpoint | 6 | 3.92% | 2.260E-04 | *MAD2L2, CDKN2A, CDKN2B, TP53, ZWINT, DDB1,* |
| GO:0012501~programmed cell death | 19 | 12.42% | 2.340E-04 | *PDCD6IP, PTEN, CDKN2A, TP53, CALR, HDAC1, HSPD1, SLC25A6, SFN, PDCD10, NCKAP1, SQSTM1, CTNNA1, PTPRC, FAF1, GSK3B, BAX, TUBB2C, VCP,* |
| GO:0051641~cellular localization | 20 | 13.07% | 4.330E-04 | *XPO1, KHDRBS1, HSP90AA1, TP53, IPO4, DDX19B, ZWINT, CALR, HSPD1, CLTA, SLC25A6, SQSTM1, NUSAP1, MYH9, FAF1, GSK3B, BAX, TUBB2C, VCP, AP3D1,* |
| GO:0006007~glucose catabolic process | 6 | 3.92% | 4.550E-04 | *ALDOA, PFKP, HK1, PGD, PGAM1, TALDO1,* |
| GO:0000086~G2/M transition of mitotic cell cycle | 4 | 2.61% | 5.130E-04 | *KHDRBS1, CDKN2A, CDKN2B, CCNB1,* |
| GO:0006066~alcohol metabolic process | 11 | 7.19% | 6.240E-04 | *ALDOA, PFKP, FDPS, HK1, PTEN, HDLBP, PGD, SREBF1, PGAM1, TALDO1, SREBF2,* |
| GO:0046907~intracellular transport | 17 | 11.11% | 6.260E-04 | *XPO1, KHDRBS1, HSP90AA1, TP53, IPO4, DDX19B, CALR, HSPD1, CLTA, SLC25A6, SQSTM1, MYH9, FAF1, GSK3B, TUBB2C, VCP, AP3D1,* |
| GO:0006281~DNA repair | 10 | 6.54% | 6.550E-04 | *TYMS, TP53, PRPF19, RUVBL2, SFPQ, XRCC6, XRCC5, VCP, CHAF1A, DDB1,* |
| GO:0051649~establishment of cellular localization | 19 | 12.42% | 8.380E-04 | *XPO1, KHDRBS1, HSP90AA1, TP53, IPO4, DDX19B, ZWINT, CALR, HSPD1, CLTA, SLC25A6, SQSTM1, NUSAP1, MYH9, FAF1, GSK3B, TUBB2C, VCP, AP3D1,* |
| GO:0051168~nuclear export | 5 | 3.27% | 8.690E-04 | *XPO1, KHDRBS1, DDX19B, GSK3B, CALR,* |
| GO:0019320~hexose catabolic process | 6 | 3.92% | 8.720E-04 | *ALDOA, PFKP, HK1, PGD, PGAM1, TALDO1,* |
| GO:0046365~monosaccharide catabolic process | 6 | 3.92% | 9.210E-04 | *ALDOA, PFKP, HK1, PGD, PGAM1, TALDO1,* |
| GO:0009719~response to endogenous stimulus | 12 | 7.84% | 9.730E-04 | *SFN, TYMS, TP53, PRPF19, RUVBL2, SFPQ, BAX, XRCC6, XRCC5, VCP, CHAF1A, DDB1,* |
| GO:0009056~catabolic process | 17 | 11.11% | 9.980E-04 | *ACLY, CDKN2A, IPO4, HNRPD, PFKP, ALDOA, HK1, RNASEH2A, PGD, SQSTM1, AFG3L2, YME1L1, FAF1, BAX, PGAM1, TALDO1, VCP,* |
| GO:0046164~alcohol catabolic process | 6 | 3.92% | 1.024E-03 | *ALDOA, PFKP, HK1, PGD, PGAM1, TALDO1,* |
| GO:0042981~regulation of apoptosis | 14 | 9.15% | 1.049E-03 | *PTEN, CDKN2A, TP53, CALR, HDAC1, HSPD1, SFN, CTNNA1, PTPRC, GSK3B, BAX, FAF1, TUBB2C, VCP,* |
| GO:0000079~regulation of cyclin-dependent protein kinase activity | 5 | 3.27% | 1.094E-03 | *SFN, GTPBP4, PTEN, CDKN2A, CDKN2B,* |
| GO:0043067~regulation of programmed cell death | 14 | 9.15% | 1.159E-03 | *PTEN, CDKN2A, TP53, CALR, HDAC1, HSPD1, SFN, CTNNA1, PTPRC, GSK3B, BAX, FAF1, TUBB2C, VCP,* |
| GO:0044265~cellular macromolecule catabolic process | 11 | 7.19% | 1.229E-03 | *HNRPD, ALDOA, PFKP, HK1, RNASEH2A, PGD, SQSTM1, FAF1, PGAM1, TALDO1, VCP,* |
| GO:0006461~protein complex assembly | 10 | 6.54% | 1.320E-03 | *XPO1, CLTA, TP53, IPO4, CTNNA1, FAF1, BAX, AP3D1, CHAF1A, CAPG,* |
| GO:0043281~regulation of caspase activity | 5 | 3.27% | 1.356E-03 | *SFN, CDKN2A, TP53, BAX, VCP,* |
| GO:0051716~cellular response to stimulus | 4 | 2.61% | 1.383E-03 | *CDKN2B, TP53, SREBF1, GSK3B,* |
| GO:0006259~DNA metabolic process | 18 | 11.76% | 1.679E-03 | *CDKN2A, TP53, NASP, HP1BP3, SFPQ, XRCC5, HDAC1, DDB1, CHAF1A, GTPBP4, SMARCA4, TYMS, RNASEH2A, PRPF19, RUVBL2, BAX, XRCC6, VCP,* |
| GO:0006913~nucleocytoplasmic transport | 7 | 4.58% | 1.775E-03 | *XPO1, KHDRBS1, IPO4, DDX19B, FAF1, GSK3B, CALR,* |
| GO:0016071~mRNA metabolic process | 10 | 6.54% | 1.856E-03 | *HNRPD, HNRPH3, YBX1, HNRPF, KHDRBS1, PABPC1, DHX15, PRPF19, SFPQ, EFTUD2,* |
| GO:0051169~nuclear transport | 7 | 4.58% | 1.905E-03 | *XPO1, KHDRBS1, IPO4, DDX19B, FAF1, GSK3B, CALR,* |
| GO:0006397~mRNA processing | 9 | 5.88% | 2.315E-03 | *HNRPH3, YBX1, HNRPF, KHDRBS1, PABPC1, DHX15, PRPF19, SFPQ, EFTUD2,* |
| GO:0044248~cellular catabolic process | 14 | 9.15% | 2.597E-03 | *ACLY, CDKN2A, HNRPD, PFKP, ALDOA, HK1, RNASEH2A, SQSTM1, PGD, BAX, PGAM1, FAF1, TALDO1, VCP,* |
| GO:0044275~cellular carbohydrate catabolic process | 6 | 3.92% | 3.038E-03 | *ALDOA, PFKP, HK1, PGD, PGAM1, TALDO1,* |
| GO:0007093~mitotic cell cycle checkpoint | 4 | 2.61% | 3.114E-03 | *MAD2L2, CDKN2B, TP53, ZWINT,* |
| GO:0033036~macromolecule localization | 17 | 11.11% | 3.329E-03 | *PDCD6IP, XPO1, KHDRBS1, TP53, IPO4, NASP, DDX19B, CALR, HSPD1, CLTA, SQSTM1, MYH9, FAF1, GSK3B, BAX, VCP, AP3D1,* |
| GO:0022408~negative regulation of cell-cell adhesion | 3 | 1.96% | 3.391E-03 | *GTPBP4, PTEN, CDKN2A,* |
| GO:0031669~cellular response to nutrient levels | 3 | 1.96% | 3.391E-03 | *CDKN2B, TP53, SREBF1,* |
| GO:0007088~regulation of mitosis | 5 | 3.27% | 3.523E-03 | *MAD2L2, CDKN2B, TP53, ZWINT, NUSAP1,* |
| GO:0019318~hexose metabolic process | 7 | 4.58% | 3.626E-03 | *ALDOA, PFKP, HK1, PTEN, PGD, PGAM1, TALDO1,* |
| GO:0016052~carbohydrate catabolic process | 6 | 3.92% | 3.958E-03 | *ALDOA, PFKP, HK1, PGD, PGAM1, TALDO1,* |
| GO:0006006~glucose metabolic process | 6 | 3.92% | 4.104E-03 | *ALDOA, PFKP, HK1, PGD, PGAM1, TALDO1,* |
| GO:0005996~monosaccharide metabolic process | 7 | 4.58% | 4.195E-03 | *ALDOA, PFKP, HK1, PTEN, PGD, PGAM1, TALDO1,* |
| GO:0008380~RNA splicing | 8 | 5.23% | 4.264E-03 | *HNRPH3, YBX1, HNRPF, PABPC1, DHX15, PRPF19, SFPQ, EFTUD2,* |
| GO:0006919~caspase activation | 4 | 2.61% | 4.321E-03 | *CDKN2A, TP53, BAX, VCP,* |
| GO:0008104~protein localization | 16 | 10.46% | 4.594E-03 | *PDCD6IP, XPO1, IPO4, TP53, NASP, DDX19B, CALR, HSPD1, CLTA, SQSTM1, MYH9, FAF1, GSK3B, BAX, VCP, AP3D1,* |
| GO:0009892~negative regulation of metabolic process | 11 | 7.19% | 5.711E-03 | *SPTBN1, GTPBP4, KHDRBS1, SMARCA4, CDKN2A, CDKN2B, TP53, ILF3, MAP4, PHB2, CAPG,* |
| GO:0022407~regulation of cell-cell adhesion | 3 | 1.96% | 5.776E-03 | *GTPBP4, PTEN, CDKN2A,* |
| GO:0043280~positive regulation of caspase activity | 4 | 2.61% | 6.181E-03 | *CDKN2A, TP53, BAX, VCP,* |
| GO:0008632~apoptotic program | 5 | 3.27% | 6.192E-03 | *SFN, CDKN2A, TP53, BAX, VCP,* |
| GO:0051301~cell division | 8 | 5.23% | 6.423E-03 | *MAD2L2, CCDC5, NUDC, NDE1, ZWINT, NUSAP1, MYH9, CCNB1,* |
| GO:0031668~cellular response to extracellular stimulus | 3 | 1.96% | 6.699E-03 | *CDKN2B, TP53, SREBF1,* |
| GO:0007050~cell cycle arrest | 5 | 3.27% | 6.739E-03 | *KHDRBS1, CDKN2A, CDKN2B, TP53, EIF4G2,* |
| GO:0009607~response to biotic stimulus | 9 | 5.88% | 7.036E-03 | *HSPD1, HSPA4, XPO1, HSP90AA1, TP53, PTPRC, GSK3B, VCP, TRAP1,* |
| GO:0048468~cell development | 21 | 13.73% | 7.197E-03 | *PDCD6IP, PTEN, CDKN2A, TP53, CALR, EIF4G2, HDAC1, HSPD1, SLC25A6, SFN, SMARCA4, PDCD10, NCKAP1, CTNNA1, SQSTM1, PTPRC, FAF1, GSK3B, BAX, TUBB2C, VCP,* |
| GO:0006950~response to stress | 19 | 12.42% | 7.549E-03 | *HSPA4, HSP90AA1, TP53, SREBF1, SFPQ, XRCC5, TRAP1, DDB1, CHAF1A, HSPD1, SFN, TYMS, PRPF19, RUVBL2, SQSTM1, GSK3B, BAX, XRCC6, VCP,* |
| GO:0022607~cellular component assembly | 13 | 8.50% | 7.623E-03 | *XPO1, IPO4, TP53, HP1BP3, EIF4G2, CHAF1A, CAPG, CLTA, SMARCA4, CTNNA1, BAX, FAF1, AP3D1,* |
| GO:0043065~positive regulation of apoptosis | 8 | 5.23% | 7.768E-03 | *SFN, PTEN, CDKN2A, TP53, PTPRC, FAF1, BAX, TUBB2C,* |
| GO:0043068~positive regulation of programmed cell death | 8 | 5.23% | 8.093E-03 | *SFN, PTEN, CDKN2A, TP53, PTPRC, FAF1, BAX, TUBB2C,* |
| GO:0006839~mitochondrial transport | 4 | 2.61% | 9.482E-03 | *HSPD1, SLC25A6, HSP90AA1, TP53,* |
| GO:0033554~cellular response to stress | 3 | 1.96% | 9.839E-03 | *TP53, SREBF1, GSK3B,* |
| GO:0051789~response to protein stimulus | 5 | 3.27% | 9.950E-03 | *HSPD1, HSPA4, HSP90AA1, VCP, TRAP1,* |
| GO:0006986~response to unfolded protein | 5 | 3.27% | 9.950E-03 | *HSPD1, HSPA4, HSP90AA1, VCP, TRAP1,* |
| GO:0006886~intracellular protein transport | 10 | 6.54% | 1.045E-02 | *HSPD1, XPO1, CLTA, IPO4, DDX19B, FAF1, GSK3B, CALR, VCP, AP3D1,* |
| GO:0006611~protein export from nucleus | 3 | 1.96% | 1.100E-02 | *XPO1, GSK3B, CALR,* |
| GO:0045786~negative regulation of progression through cell cycle | 7 | 4.58% | 1.114E-02 | *KHDRBS1, PTEN, CDKN2A, CDKN2B, TP53, BAX, EIF4G2,* |
| GO:0051325~interphase | 5 | 3.27% | 1.147E-02 | *KHDRBS1, CDKN2A, CDKN2B, PTPRC, CCNB1,* |
| GO:0065003~macromolecular complex assembly | 12 | 7.84% | 1.161E-02 | *XPO1, CLTA, TP53, IPO4, CTNNA1, HP1BP3, FAF1, BAX, AP3D1, EIF4G2, CHAF1A, CAPG,* |
| GO:0008283~cell proliferation | 15 | 9.80% | 1.162E-02 | *NUDC, PTEN, KHDRBS1, CDKN2B, CDKN2A, TP53, NASP, IMPDH2, ZNF259, GTPBP4, SFN, CTNNA1, PTPRC, BAX, CAPNS1,* |
| GO:0043285~biopolymer catabolic process | 9 | 5.88% | 1.164E-02 | *HNRPD, CDKN2A, RNASEH2A, AFG3L2, SQSTM1, YME1L1, FAF1, BAX, VCP,* |
| GO:0048523~negative regulation of cellular process | 19 | 12.42% | 1.183E-02 | *KHDRBS1, PTEN, CDKN2A, CDKN2B, TP53, MAP4, EIF4G2, HDAC1, CAPG, SPTBN1, GTPBP4, SMARCA4, ILF3, CTNNA1, PTPRC, FAF1, GSK3B, BAX, PHB2,* |
| GO:0006917~induction of apoptosis | 7 | 4.58% | 1.214E-02 | *SFN, PTEN, CDKN2A, TP53, PTPRC, BAX, TUBB2C,* |
| GO:0008637~apoptotic mitochondrial changes | 3 | 1.96% | 1.223E-02 | *SFN, CDKN2A, BAX,* |
| GO:0012502~induction of programmed cell death | 7 | 4.58% | 1.240E-02 | *SFN, PTEN, CDKN2A, TP53, PTPRC, BAX, TUBB2C,* |
| GO:0044262~cellular carbohydrate metabolic process | 9 | 5.88% | 1.323E-02 | *ALDOA, PFKP, HK1, PTEN, ACLY, PGD, PGAM1, GSK3B, TALDO1,* |
| GO:0045184~establishment of protein localization | 14 | 9.15% | 1.576E-02 | *PDCD6IP, XPO1, IPO4, NASP, DDX19B, CALR, HSPD1, CLTA, MYH9, GSK3B, BAX, FAF1, VCP, AP3D1,* |
| GO:0048522~positive regulation of cellular process | 17 | 11.11% | 1.592E-02 | *PTEN, CDKN2B, CDKN2A, TP53, SFN, ILF3, SQSTM1, EEF1D, CTNNA1, PTPRC, NUSAP1, FAF1, GSK3B, BAX, XRCC6, CAPNS1, TUBB2C,* |
| GO:0051252~regulation of RNA metabolic process | 3 | 1.96% | 1.623E-02 | *HNRPD, HNRPF, PABPC1,* |
| GO:0009058~biosynthetic process | 23 | 15.03% | 1.691E-02 | *MRPS2, NQO1, ACLY, HSP90AA1, ATP5A1, FPGS, IMPDH2, ALDH18A1, SARS, EIF4G2, NARS, FDPS, TYMS, RPL6, EEF1D, PTPRC, IARS, AARS, AIPL1, MGAT4B, AP3D1, EEF2, MRPL37,* |
| GO:0045859~regulation of protein kinase activity | 7 | 4.58% | 1.714E-02 | *SFN, GTPBP4, PTEN, CDKN2A, CDKN2B, PTPRC, GNB2L1,* |
| GO:0048519~negative regulation of biological process | 19 | 12.42% | 1.758E-02 | *KHDRBS1, PTEN, CDKN2A, CDKN2B, TP53, MAP4, EIF4G2, HDAC1, CAPG, SPTBN1, GTPBP4, SMARCA4, ILF3, CTNNA1, PTPRC, FAF1, GSK3B, BAX, PHB2,* |
| GO:0006984~ER-nuclear signaling pathway | 3 | 1.96% | 1.768E-02 | *TP53, GSK3B, VCP,* |
| GO:0006096~glycolysis | 4 | 2.61% | 1.790E-02 | *ALDOA, PFKP, HK1, PGAM1,* |
| GO:0043549~regulation of kinase activity | 7 | 4.58% | 1.920E-02 | *SFN, GTPBP4, PTEN, CDKN2A, CDKN2B, PTPRC, GNB2L1,* |
| GO:0048518~positive regulation of biological process | 18 | 11.76% | 1.938E-02 | *PTEN, CDKN2B, HSP90AA1, CDKN2A, TP53, SFN, ILF3, SQSTM1, EEF1D, CTNNA1, PTPRC, NUSAP1, FAF1, GSK3B, BAX, XRCC6, CAPNS1, TUBB2C,* |
| GO:0043039~tRNA aminoacylation | 4 | 2.61% | 1.947E-02 | *NARS, IARS, AARS, SARS,* |
| GO:0043038~amino acid activation | 4 | 2.61% | 1.947E-02 | *NARS, IARS, AARS, SARS,* |
| GO:0006418~tRNA aminoacylation for protein translation | 4 | 2.61% | 1.947E-02 | *NARS, IARS, AARS, SARS,* |
| GO:0051338~regulation of transferase activity | 7 | 4.58% | 2.104E-02 | *SFN, GTPBP4, PTEN, CDKN2A, CDKN2B, PTPRC, GNB2L1,* |
| GO:0008285~negative regulation of cell proliferation | 7 | 4.58% | 2.142E-02 | *GTPBP4, PTEN, CDKN2A, CDKN2B, TP53, CTNNA1, BAX,* |
| GO:0015031~protein transport | 13 | 8.50% | 2.200E-02 | *PDCD6IP, XPO1, IPO4, NASP, DDX19B, CALR, HSPD1, CLTA, MYH9, GSK3B, FAF1, AP3D1, VCP,* |
| GO:0030155~regulation of cell adhesion | 4 | 2.61% | 2.283E-02 | *GTPBP4, PTEN, CDKN2A, FAF1,* |
| GO:0005975~carbohydrate metabolic process | 11 | 7.19% | 2.359E-02 | *ALDOA, PFKP, HK1, PTEN, ACLY, PGD, PGAM1, GSK3B, TALDO1, MGAT4B, GANAB,* |
| GO:0051345~positive regulation of hydrolase activity | 4 | 2.61% | 2.372E-02 | *CDKN2A, TP53, BAX, VCP,* |
| GO:0007162~negative regulation of cell adhesion | 3 | 1.96% | 2.396E-02 | *GTPBP4, PTEN, CDKN2A,* |
| GO:0006399~tRNA metabolic process | 5 | 3.27% | 2.435E-02 | *NARS, MTO1, IARS, AARS, SARS,* |
| GO:0055086~nucleobase, nucleoside and nucleotide metabolic process | 7 | 4.58% | 2.507E-02 | *ACLY, TYMS, ATP5A1, PGD, FPGS, IMPDH2, TALDO1,* |
| GO:0044249~cellular biosynthetic process | 18 | 11.76% | 2.827E-02 | *MRPS2, NQO1, HSP90AA1, ATP5A1, FPGS, IMPDH2, ALDH18A1, SARS, EIF4G2, NARS, TYMS, RPL6, EEF1D, IARS, AARS, AP3D1, MRPL37, EEF2,* |
| GO:0008203~cholesterol metabolic process | 4 | 2.61% | 3.043E-02 | *FDPS, HDLBP, SREBF1, SREBF2,* |
| GO:0050790~regulation of catalytic activity | 10 | 6.54% | 3.078E-02 | *SFN, GTPBP4, PTEN, CDKN2A, CDKN2B, TP53, PTPRC, BAX, GNB2L1, VCP,* |
| GO:0016567~protein ubiquitination | 4 | 2.61% | 3.252E-02 | *GTPBP4, PRPF19, SAE1, VCP,* |
| GO:0046822~regulation of nucleocytoplasmic transport | 3 | 1.96% | 3.290E-02 | *KHDRBS1, FAF1, GSK3B,* |

*Generic biological processes (% > 25) were removed from the table.
